# Feasibility of trauma-focused cognitive behavioural therapy for patients with PTSD and psychosis

**DOI:** 10.3389/fpsyt.2024.1375972

**Published:** 2024-10-24

**Authors:** Caecilie B. Buhmann, Erik Lykke Mortensen, Frida Lau Graebe, Sarah K. Larsen, Susanne Harder, Sidse Arnfred, Stephen F. Austin

**Affiliations:** ^1^ Psychiatric Research Unit, Region Zealand, Slagelse, Denmark; ^2^ Department of Psychology, Institute for Psychology, University of Copenhagen, Copenhagen, Denmark; ^3^ Department of Public Health, University of Copenhagen, Copenhagen, Denmark; ^4^ Mental Health Centre North Zealand, Mental Health Services, Hillerod, Capital Region of Denmark, Denmark; ^5^ Department of Clinical Medicine, Faculty of Health and Medical Sciences, Copenhagen University, Copenhagen, Denmark

**Keywords:** PTSD, Trauma-Focused Cognitive Behavioral Therapy, severe mental illness, psychosis, feasibility

## Abstract

Studies have shown a high prevalence of trauma and PTSD among patients with severe mental illness, but relatively few studies have examined the outcomes of PTSD treatment for this patient group. The aim of this case-series was to assess the feasibility of a Trauma-Focused Cognitive Behavioral Therapy (TF-CBT) intervention for PTSD in people with psychosis. The study examined the possibilities and obstacles when treating this population within clinical settings. Patients were selected from four community mental health centers and were screened for traumatic experiences and symptoms of PTSD. A small group of eligible participants (n=7) received manualized TF-CBT adapted for patients with psychosis. Experienced therapists received training and supervision in the intervention. Symptoms of PTSD and psychosis were assessed at baseline and post-treatment along with quality of life, level of functioning, alliance, life events, engagement, suicidal ideation and adverse events. Treatment fidelity and the different combinations of treatment modules were monitored in regard to implementation. Three cases were selected as illustrative of the different treatment courses when implementing the TF-CBT intervention within this population. Detailed case descriptions were based on quantitative ratings and the therapists’ experiences with the therapy. Results from the case series highlighted issues regarding toleration of treatment, large variation in psychopathology and the task of matching treatment needs with appropriate therapeutic techniques. The complexity of the patient group may affect treatment and clinical research studies. Lessons learned from this case series can contribute to the future development implementation and evaluation of trauma treatment for patients with psychosis.

## 1 Introduction

Patients with severe mental illness (SMI) have high risk of traumatic experiences and an increased vulnerability to develop PTSD after trauma (1). The prevalence of trauma exposure in this patient group ranges from 49 to 100% and PTSD can be as prevalent as 50% in patients with comorbid psychosis (2, 3) albeit under-diagnosed (1, 4). There is a clear positive correlation between psychosis and co-morbid PTSD with increased cognitive, affective and behavioral disturbances (5) and the social and psychological consequences of comorbid SMI and PTSD or trauma can be severe, resulting in aggravation of illness and treatment complications (1). Patients with SMI are rarely offered trauma-focused treatment (2, 6, 7), although it is well established that trauma plays a role in the onset of schizophrenia and other psychoses (8).

There is well-established evidence for the effects of psychotherapy for PTSD including Eye Movement Desensitization and Reprocessing Therapy (EMDR) and Trauma-Focused Cognitive Behavioral Therapy (TF-CBT) such as Prolonged Exposure (PE), Cognitive Processing Therapy (CPT) and various other TF-CBT protocols with varying emphasis on cognitive or behavioral elements (9, 10)., Despite the range of established interventions for PTSD there is still a limited evidence for PTSD treatment in patients with psychosis (11). One concern with PTSD treatment in patients with co-morbid psychosis is that treatment will exacerbate both symptoms of PTSD and psychosis potentially resulting in high drop-out rates (7, 12, 13). Another concern is whether the complexity of treatment for these patients is possible in standard clinical settings. Several studies investigating the effects of various trauma therapies for patients with comorbid PTSD and psychosis suggest that it can be both safe and effective (11, 14, 15), although study populations have been diagnostically heterogeneous. Effectiveness of cognitive behavioral therapy (CBT) has been investigated in several RCT’s (16–18). In two studies, CBT showed significant effect in comparison with TAU (18) and an active control condition (16), while Steel et al. Study (17) found no differences between CBT and TAU on PTSD symptoms. The treatment protocols were adapted from standard TF-CBT with less focus on trauma exposure and more emphasis on psychoeducation and cognitive restructuring (19) Van der Berg et al. (20) found significant effect of both EMDR and Prolonged Exposure (PE) in comparison to a waiting list group on measures of PTSD symptoms, while EMDR and PE were equally effective. Recently, two studies have contributed to the promising effect of EMDR. Marlow et al. (21) found significant effect of EMDR compared with TAU in an exploratory RCT on measures of PTSD symptoms, while Varese et al. (22) reported promising results of EMDR adapted for psychosis in comparison with TAU in a feasibility RCT. A Cochrane review and two systematic reviews on PE and all trauma-focused therapies for patients with PTSD and psychosis concluded that results were inconclusive and that further trials are needed to confirm or refute the preliminary findings (11, 23, 24).

One reason why these studies are inconclusive may be related to issues of feasibility of conducting research with this population due to highly complex psychopathology. There are a number of ongoing trials in this area (13, 16). Psychotherapy for trauma for people with psychosis is complicated and it is pertinent to explore the feasibility of providing this treatment within a routine clinical setting.

In a Danish public health care context under resource constraints, such treatment would only be offered in specialist clinics with access for a selected small group of patients but given the prevalence of trauma in patients with psychosis, it is important to make trauma treatment more available in a community mental health setting. Treatment in community health services usually includes regular contact with a nurse every 1-12 weeks, appointments with a medical doctor when adjustments in pharmacotherapy is needed and in rare instances supportive psychotherapy or psychoeducation with a psychologist. CBT has been implemented widely in mental health care in Denmark by staff with various backgrounds and training. Thus a decision to use a, TF-CBT intervention inspired by models by Clark and Ehlers (25) was judged to be a feasible solution and was less demanding on resources and staff training than PE or EMDR.

The aim of this pilot study was to assess the feasibility of a new TF-CBT protocol for PTSD in people with psychosis. The study examined the possibilities and obstacles arising when treating this population within a community mental health setting, including the acceptability of the treatment, engagement and a range of internal and external factors related to treatment.

## 2 Context

### 2.1 Study design and study population

The case series was an open clinical trial where patients were assessed at baseline and post-treatment by independent raters with psychiatric training and followed on a number of parameters during treatment. Patients from a clinical sample in community mental health services were screened clinically according to the study’s inclusion criteria based on recommendations from the patient contact persons in the community health care center.

The inclusion criteria were symptoms of PTSD measured in symptom load, relevant index trauma and the motivation to receive PTSD treatment, a schizophrenia spectrum diagnosis (ICD-10 F20 + F22-25 + F28-29), current outpatient treatment in the regional community mental health center and stable pharmacological treatment. Participants had to be more than 18 years old, provide written informed consent and be able to receive therapy in Danish. Additionally, participants should be clinically stable enough to participate in treatment, not requiring hospitalization due to risk of harm to self or others. Exclusion criteria were presence of active harmful use of alcohol or drugs (ICD-10 F1x.2 – F1x.9) and the presence of organic psychiatric disorders (ICD-10 F0-09).

### 2.2 Outcome measures

At baseline and post-treatment, PTSD was assessed using The Clinician administered PTSD scale for DSM-5 (CAPS-5) which is a 30-item structured interview (26). Trauma history was mapped using a modified version of Brief Trauma Questionnaire (BTQ), a 10-item questionnaire about the most common types of trauma, the number of times each trauma was experienced and the age at each trauma (27). The severity of psychotic symptoms was measured with Scale for Assessment of Positive Symptoms (SAPS), a 34-item interview assessing positive psychotic symptoms (28). Finally, quality of life and level of functioning was assessed using the 5-item WHO-5 self-rating (29) and Work and Social Adjustment Scale (WSAS), a 5-item rating of social functioning where a low score corresponds to higher level of functioning (30).

Throughout treatment, suicidal and self-harm behavior was monitored by asking the patient directly about whether such behavior had been present since the last rating, and if so, how many times and by which method. No formal psychometric rating was used for this purpose. Psychotic symptoms were assessed using PANSS-6, a brief 6-item observer rating developed to screen for positive and negative symptoms of schizophrenia (31). Therapeutic alliance and adverse events were measured using The Negative Events Questionnaire (NEQ), a 20-item questionnaire assessing adverse effects of psychotherapy (25, 32) and the Working Alliance Inventory (WAI), a 12-item self-rating assessing the alliance between patient and therapist, with separate self-rating questionnaires for patient and therapist. The scale is summarized in three factors; task, bond and goal with a score from 1-7 (33). Throughout the course of treatment, manual fidelity listing therapeutic interventions used in each session. Symptoms of psychosis and adverse events including self-harm and suicidal behavior were registered every eighth session of therapy.

### 2.3 CBT for PTSD in psychosis

**Figure 1:**
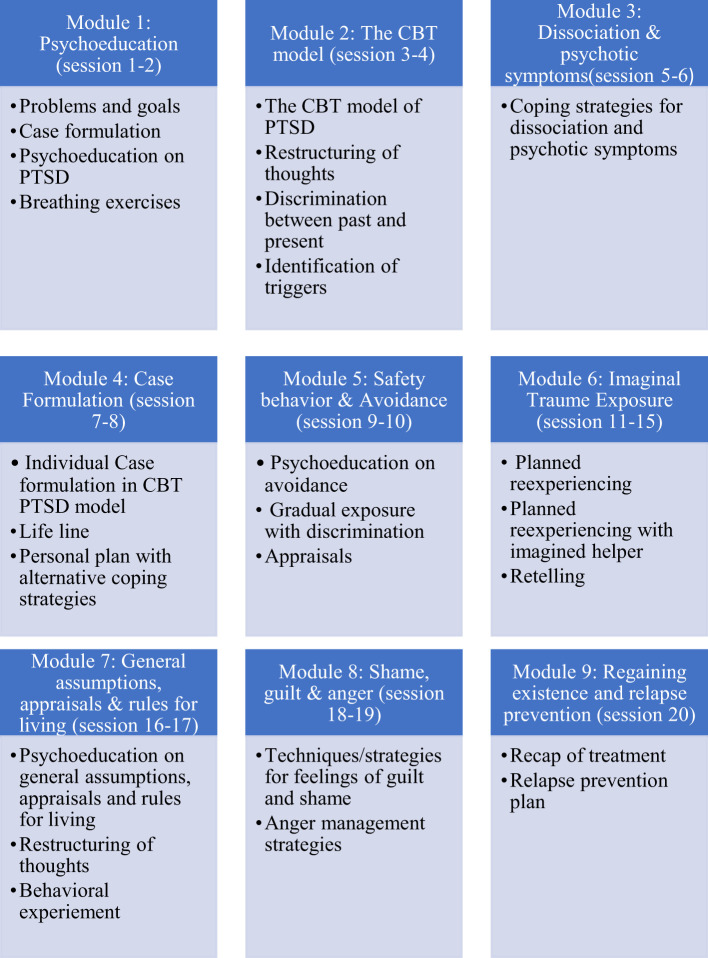
Overview of treatment manual.

A decision to adapt a TF-CBT protocol for psychosis was undertaken due to existing CBT competencies in community health care staff. Furthermore, TF-CBT [based on cognitive therapy for PTSD by Ehlers & Clark (25)] was already in use in regional psychotherapeutic PTSD clinics. Clark & Ehlers emphasize trauma exposure with update or an imaginary helper and discriminating the past and present. In the adapted version *in vivo* exposure to address avoidance, stabilizing methods such as breathing, shifts in focus of attention and coping strategies for psychotic symptoms were added. A more elaborate and individualized case formulation including PTSD and symptoms of psychosis was undertaken which included constructing a timeline of trauma exposures to reflect multiple traumas experienced by most patients. Therapy was planned to be 20-25 sessions. As standard treatment TF-CBT was 20 sessions, and it was considered appropriate to add up to 5 extra sessions to reflect treatment complexity within this population. These extra sessions could be used to promote stabilization, control and engagement or to address co-morbid issues as they arose. The protocol consisted of nine modules, which could be applied flexibly when deemed relevant by the therapist (see **Figure 1**). It was strongly recommended that the modules with psychoeducation, coping strategies, working with avoidance and trauma exposure were used with all patients, as these are core modules in TF-CBT, which was the case for all three patients in this case series. Treatment did not include working directly with psychosis, but offered some strategies for managing psychotic symptoms and addressed the individual relationship between PTSD and psychosis.

Participants continued to receive standard outpatient treatment for psychosis in the community mental health center, including psychopharmacological treatment. Six therapists, all psychologists with specialist training conducted therapy in accordance with the manual. All therapists were employed in the community mental health centers where participants received outpatient treatment for their psychosis. All therapists were experienced in delivering CBT in a psychiatric setting and had experience in providing psychotherapy to patients with psychotic disorders. Most therapists also had experience with PTSD therapy. Therapists received three days training in the TF-CBT protocol and received regular supervision from one of the authors of the treatment manual.

### 2.4 Ethics

Approval for the study was obtained from the Regional Board of Research Ethics (SJ-852) and The Danish Data Protection Agency before data collection began (REG-109-2020 and REG-110-2020). Participants provided informed consent to participate in the study. They were free to withdraw from the study at any point without any consequences to the standard care they received.

## 3 Detail

A total of 43 eligible candidates were identified, and 14 patients were consecutively invited for further assessment based on treatment capacity in the project. These patients participated in a comprehensive assessment to determine if they had PTSD based on CAPS. A total of 7 patients with clinically significant symptoms were included in the treatment. This article focused on three patients (pseudonyms) as they were seen as are representative examples of the treatment trajectories and reflective of different patient profiles. Heather had medium symptom load and fairly good level of functioning, Valerie had many cancellations, stress associated with a municipal training program and eventually a relapse into alcohol abuse, while Dorris had cognitive challenges and several hospitalizations during treatment (**Table 1**). Among the four excluded patients, the profile and outcome of one were similar to Heather’s, another had an atypical psychosis diagnosis (Non-organic psychosis, NOS) and two did not see PTSD as their main challenge, which affected their motivation for focusing on it in treatment.

**Table 1:** Overview of patients.

Name	Valerie	Heather	Doris
**Age/sex**	26/woman	30/woman	53/woman
**Diagnosis**	Schizophrenia, PTSD & ADHD	Schizophrenia & PTSD	Schizoaffective disorder & PTSD
**Substance abuse**	Cannabis & amphetamine previously. Relapse of alcohol abuse during treatment	No	No
**Psychiatric treatment**	Antipsychotic & ADHD medicines.	Antipsychotic medicine (stopped during treatment)	Antipsychotic, antidepressant and mood stabilizer
**No. of sessions**	11 (only every 4-5 weeks). Many cancelations	20 (1-4 sessions every month)	25 (2 every month) Many cognitive difficulties.
**No. of assessments during treatment**	1 time	2 times	3 times
**Trauma exposure**	Yes	No but trauma lifeline completed	Yes
**Negative events**	Moderate unpleasant memories.	Moderate increase in traumatic memories, passive suicidal thoughts and anxiety	Moderate sleep disturbance, anxiety and unpleasant feelings and memories
**Hospitalization during treatment**	No	No	Twice due to psychotic symptoms and suicidal thoughts
**Suicidal**	Previously 6 attempts. Suicidal thoughts decreased during treatment	Previously 3 attempts. Mildly distressing suicidal thoughts at the end of treatment.	Previously 1 attempt and periods with suicidal thoughts. Severe suicidal thoughts and plans during hospitalizations, but none between hospitalizations.
**Self-harm**	Regular self-harm previously. None during treatment.	Long history of self-harm. One incident during treatment.	No history of self-harm although tendency to restrict eating and excessive exercise on psychotic basis
**Alliance**	Good	Good	Good
**Life events during treatment**	Pressure from municipal work program	Many stressors related to work, economy, housing and accident of close relative at the end of treatment.	Severe illness of a close friend
**Change after treatment**	Improvement in PTSD score from high to moderate. Some improvement in function and quality of life. Psychotic symptoms are constant.	PTSD symptoms improves so that the patient does not have PTSD after treatment. Psychotic symptoms and level of functioning improve as well.	Improvement on PTSD score, PANSS-score (but not SAPS), function and quality of life.

### 3.1 Heather – a patient that successfully engaged in and completed treatment

Heather (H) was a 30-year-old married woman with paranoid schizophrenia and PTSD. She had a stable social situation with an administrative job, a house and good contact with family and friends. She suffered multiple traumas in adolescence and early adulthood, including physical and psychological violence in her childhood and from an intimate partner, two episodes of rape and being threatened at gunpoint twice. She also reported trauma from deployment in a war zone. Heather had symptoms of mental illness since the age of six and was hospitalized in her late teenage years for severe depression, which was treated with medicine and electroconvulsive therapy. The diagnosis was changed to schizophrenia at a later point and was briefly treated with antipsychotic medicine. At baseline, Heather had mild psychotic symptoms, which were mainly associated with stress. She had 3 suicide attempts, the most recent a year before enrollment in the project, and she still experienced regular suicidal thoughts. She had a history of self-harm since the age of six but had not practiced this for the past year.

Heather had 20 therapy sessions over a period of nine months corresponding to 1-4 sessions every month. She saw one therapist the first 6 sessions and another therapist for the last 14 sessions because the initial therapist left the study. However, she already knew the new therapist from the community mental health center and there were no problems in establishing a good therapeutic alliance with the new therapist.

The predominant CBT methods used included psychoeducation, identification of triggers, discrimination, coping strategies for dissociation, psychotic symptoms, cognitive restructuring and shame and guilt. Heather worked three sessions with drawing a lifeline and received psychoeducation on the rationale behind trauma exposure, but the therapist and patient decided not to prioritize the use of trauma exposure as Heather was more limited by anxiety and arousal than re-experiencing. Seven sessions were used targeting avoidance behavior using central techniques in CBT; behavioral experiments and *in vivo* exposure. A high therapeutic alliance was maintained throughout treatment with minor differences between patient and therapist (average WAI score task, bond and goal: 5,5 of 7 for the patient/4,6 for the therapist). Heather followed the course of therapy with no cancellations or failure to attend.

**Figure 2:**
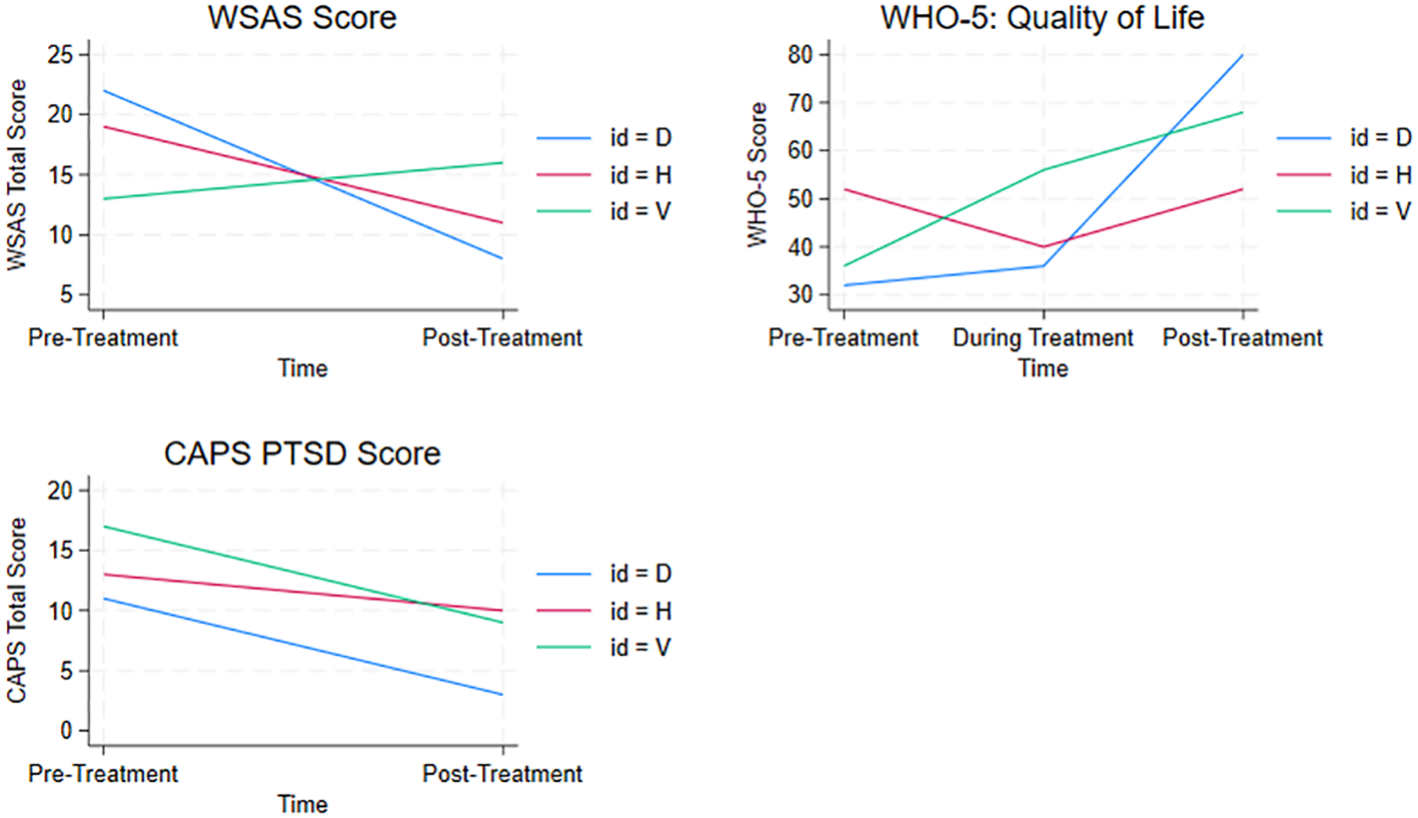
Changes total score level of functioning (WSAS low score = best level of functioning), PTSD symptoms (CAPS low score = fewest symptoms) and Quality of Life (WHO-5 high score = best quality of life) over time all three patients displayed.

At post treatment Heather did not meet the criteria for a PTSD diagnosis. Symptoms of avoidance and cognition impairment improved, whereas hypervigilance worsened during treatment and re-experiencing was constant on ratings. At post-treatment and follow-up, psychotic symptoms were unaltered from baseline to post-treatment, where symptoms were always in the “none to mild” category. Level of social functioning improved although some impairment was still present. Well-being remained constant during treatment and was within the normal range (see **Table 2** and **Figure 2**).

**Table 2:** Rating scores.

Rating	Baseline	Post-treatment	Baseline	Post-treatment	Baseline	Post-treatment
CAPS						
PTSD (yes – no)	Yes	No	Yes	Yes	No	No
PTSD intensity score (0-80)	41	33	51	23	31	8
Avoidance (0-5)	1	0	2	2	1	1
Reexperiencing (0-2)	4	4	5	4	4	1
Cognition (0-7)	7	3	6	1	6	0
Hypervigility (0-6)	2	4	4	2	0	1
CAPS Total (0-20)	14	11	17	9	11	3
SAPS						
Hallucinations (0-30)	1	2	2	3	1	2
Delusions (0-60)	2	2	2	1	0	0
Bizarre behavior (0-20)	0	1	0	0	0	0
Thought disorder (0-40)	2	0	0	0	0	0
**PANSS-6 (0-36)**	15	–	14	–	16	3
**WSAS (0-40)**	25	17	13	21	22	8
**WHO-5 (0-100)**	68	68	36	68	32	80

CAPS, SAPS, PANSS and WSAS the lower score, the better. WHO-5 the higher score, the better.

Heather reported a temporary moderate increase in restlessness, re-experiencing memories of past events and thoughts of not wanting to be alive in the first part of treatment. At this time, she also reported numerous stressors related to her economy and housing and a close relation being in a serious accident. Midway through treatment Heather reported moderate anxiety and restlessness associated with the treatment, not wanting to be alive and fear that she would not get better. Heather reported one incident of self-harm hitting herself after the first session. During treatment, she was not suicidal at any point and was not hospitalized. Heather stopped taking antipsychotic medication during treatment and did not experience hallucinations or delusions.

In summary, Heather represents a patient, who had a high level of functioning and discrete psychotic symptoms and engaged well in treatment. She recovered from PTSD and reported no severe adverse events. It is noteworthy that the therapy course did not entail direct trauma exposure.

### 3.2 Valerie-a patient that experienced challenges engaging in treatment

Valerie was a 26-year-old woman with schizophrenia, PTSD and ADHD. She lived with her partner, but otherwise had a limited social network, mainly her family. She was unemployed, received social benefits and had no education beyond secondary school. Valerie had suffered multiple traumas as a child and adult. Her father had sexually abused her throughout her childhood into young adulthood. Furthermore, she was sexually assaulted as a young adult, had been in a verbally and physically abusive relationship around the same time and lost a close friend in an accident. Her psychiatric history included several depressive episodes, different treatment courses for eating disorder, long-term cannabis and amphetamine abuse and multiple suicide attempts between the ages of 13-18. She had the first psychotic episode when she was 15. Her current psychotic symptoms included auditory hallucinations, tactile hallucinations, and paranoid delusions. Many of her psychotic experiences were trauma-related in content. She suffered from trauma-related avoidance to such an extent that it had resulted in social isolation. Valerie engaged in self-harm behavior when she enrolled in the research project. At the initial screening, she was assessed as being in a stable period of her illness without addictive behavior. Valerie received ADHD and antipsychotic medication at the beginning of treatment and there were no changes in medication during treatment.

Valerie received only 11 treatment sessions with treatment taking place every 4-5 weeks, because she had many cancellations and an unstable attendance, and she stopped the treatment earlier than planned due to non-attendance. The therapist made several efforts to continue treatment with the patient. During treatment, Valerie was in a municipal program to clarify her ability to work, which she experienced as a stressor interfering with treatment. For a few months, attendance was stable, coinciding with a break in the municipal employment program. The instability returned in the last 2 months, coinciding with a relapse to alcohol abuse. Until the frequent cancellations began, the therapist and patient both evaluated the therapeutic alliance as good (average WAI score for task, bond and goal 6,5 of 7 possible for the patient and 5 for the therapist).

The therapeutic methods predominantly used were psychoeducation, discrimination, identification of triggers, diversion techniques to reduce hyperarousal and dissociation. The patient worked with *in vivo* exposure over four sessions, which resulted in less social isolation and trauma exposure over three sessions in the form of exposure with an imaginary helper (imagery rescripting). In relation to trauma exposure, there was a slight deterioration in Valerie’s condition, but she attributed this to stress associated with the employment program and not treatment. She reported that hallucinations and delusions were less frequent after treatment. The therapist suggested that Valerie would have benefitted more from therapy if stress related to the municipal work ability program had been avoided.

Valerie improved slightly on re-experiencing and hypervigilance after treatment and PTSD scores decreased, but the patient still met the criteria for PTSD after treatment. Psychotic symptoms remained more or less constant, mostly in the mild category. Level of functioning indicated significant functional impairment which did not improve after treatment. There was a significant improvement in well-being from poor levels to within normal levels after treatment (see **Table 2** and **Figure 2**).

Valerie was not hospitalized during treatment and did not have suicidal or self-harming behavior and the distress associated with suicidal thoughts decreased. After 8 sessions, Valerie reported being moderately affected by re-experiencing traumatic events and increased stress/hyperarousal. She associated these problems with the treatment. She also reported a moderate increase in restlessness due to conflicts with her family and her case at the municipality.

In summary, Valerie represented a patient who had difficulties engaging in treatment, and consequently had a shorter course of treatment. Although therapy was stopped prematurely both Valerie and the therapist reported a positive effect of treatment, which was however only partially mirrored on the assessment scores. This case raises an issue of whether the harmful coping and early termination of treatment was due to treatment itself or other stressful factors in the patient’s life. Valerie worked directly with trauma exposure during treatment and was able to tolerate it, experiencing positive effects although she reported temporary increase in some symptoms.

### 3.3 Doris – a patient who completed treatment despite challenges

Doris (D) was a 53-year-old married woman with PTSD and Schizoaffective Psychosis of the depressive type who received early pension due to onset of psychosis. Doris and her spouse had a good network of friends, but she had a strained relationship with her stepfather and sister. Doris experienced multiple traumas in childhood and adolescence, including physical and sexual abuse by caregivers to the point of fearing for her life on multiple occasions. She had further suffered two incidents of sexual assault in adulthood.

Since adolescence, Doris had numerous hospitalizations due to psychotic symptoms and depressive episodes leading to suicidal thoughts and plans, restrictive eating and excessive exercise. The hospitalizations were typically related to external stressors such as her own illness as well as friends’ and family illness. Doris reported one suicide attempt by overdose more than ten years prior to enrolling in the project, but she had no history of other self-harm.

Doris received 25 therapy sessions over a period of 16 months, typically 2 sessions every month, although the frequency was less at the end of treatment due to hospitalization. She was punctual with few cancellations. The psychopharmacological treatment included antipsychotic, antidepressant and mood stabilizing medicines. The antipsychotic drug was switched to another during treatment.

Doris experienced cognitive impairment after electroconvulsive treatment administered before the psychotherapy started and often forgot the content of sessions. This difficulty could have potentially influenced her reporting of symptoms, adverse events and life events.

Doris utilized the majority of the techniques in the manual, but in particular psychoeducation, discrimination, identification of triggers, coping strategies for dissociation, hyperarousal and psychotic symptoms, cognitive restructuring and *in vivo* exposure. Doris worked with avoidance in eight sessions and imaginary trauma exposure in one session. Due to her cognitive impairment, Doris received written cards with the most important points from therapy to assist memory and integration of learning, which seemed to work well. The therapeutic alliance was characterized by trust, sympathy and respect (average WAI score task, bond and goal 6,5 of 7 possible for the patient and 5 for the therapist). Doris and the therapist knew each other from previous hospitalization, which the therapist described as an advantage because Doris was very reluctant to open up to new people. Once during therapy, when planning trauma exposure, Doris considered stopping, but trauma exposure was later successfully undertaken.

Doris reported minimal to mild psychotic symptoms at the commencement of treatment and these symptoms were relatively stable throughout treatment. There was a slight decrease in overall psychopathology. Doris only had a moderate PTSD score at baseline and did not formally meet criteria for a PTSD diagnosis due to low levels of hyperarousal. After treatment, she displayed practically no symptoms of PTSD. Symptoms of psychosis were also low and improved during treatment. Well-being and level of functioning also improved (see **Table 2** and **Figure 2**). At post treatment, she reported no suicidal thoughts or plans.

Doris reported several time-limited negative adverse events, which she associated with the treatment, including sleeping problems, anxiety, and having unpleasant emotions and memories resurfacing. During treatment, a friend suffered from serious illness, which Doris reported as a possible contributing factor to worsening in her condition. She was hospitalized twice during the course of therapy due to suicidal thoughts and psychosis, but this was not reflected in the questionnaires monitoring suicidal thoughts. The therapist explained that even the day before hospitalization Doris had not reported suicidal symptoms over the telephone and symptoms were not present after hospitalization. In supervision, the therapist decided to continue supportive therapy during the hospitalizations in close collaboration with the staff at the bed ward. The therapist found that external stressors and negative basic assumptions destabilized the patient, and that the hospitalizations were not directly related to therapy. However, it could not be ruled out that trauma exposure was a contributing factor to the reactivation of psychosis and depressive symptoms. According to the therapist’s assessment psychotic symptoms and suicidal ideation did not directly influence the PTSD treatment, as Doris was in a stable state after each discharge. The therapist reported that Doris explicitly mentioned having experienced the therapy as meaningful and helpful despite the adverse events associated with therapy and the patient’s fear of doing *in vivo* and imaginary exposure. This is reflected in the outcome scores.

In summary, Doris was a patient with co-occurring symptoms of PTSD and psychosis, and a relatively low level of symptoms on ratings, but she periodically experienced high distress, that resulted in recurring hospitalizations. However, she felt supported by therapy and was able to engage in trauma exposure without there being strong links between this and worsening of symptoms.

## 4 Discussion

The case series examined the implementation of psychotherapy for PTSD for patients with co-morbid psychosis within a routine clinical setting. The three cases highlighted the complexity of the mental health and social problems experienced by participants and the variability in treatment course and outcomes. While the treatment courses were characterized by challenges including external stressors, hospitalizations, cognitive difficulties, relapse of substance abuse, regular self-harm, suicidal thoughts and competing comorbidities, all three patients showed some degree of improvement after treatment.

### 4.1 Toleration of treatment including exposure

All three patients seemed to tolerate therapy based on ratings on adverse events and low dropout. All patients reported a short-term increase in PTSD-like symptoms such as increased awareness of memories of the past, increased restlessness, hyperarousal and anxiety. However, this reduced over time which is in line with Burger et al. study which concluded that symptom exacerbation among patients with PTSD and psychosis receiving treatment for their trauma is relatively common but unrelated to poor treatment responses or increased risk of drop-out (12).

Two patients had severely unhealthy coping strategies including excessive exercise, restrictive eating, and substance abuse and experienced suicidal thoughts as a response to emotional distress. One patient (Valerie) relapsed to alcohol abuse, and the other patient (Doris) occasionally utilized maladaptive coping mechanisms in times of distress.

Doris was hospitalized twice during treatment, but each time returned to therapy. Although she identified the treatment as a contributing factor in PTSD symptom exacerbation, neither she nor therapist judged the trauma therapy to be the direct cause of the two hospitalizations. It is difficult, if not impossible, to evaluate whether reported worsening was due to therapy or other factors, since all participants experienced negative life events during treatment, some of traumatic intensity and others as general stressors. All patients and therapists identified external factors as an important contributing reason for exacerbation in patient symptoms.

There was no worsening of psychotic symptoms during or after treatment in the other two patients (Heather & Valerie). However, all three patients generally scored low on psychotic symptoms throughout treatment. The majority of published studies, evaluating PTSD treatment for patients with comorbid psychosis, have found that psychotic symptoms neither decrease nor increase during or after treatment (7, 16, 17, 20, 34, 35). One study (36) found a significant reduction in psychotic symptoms such as hallucinations and paranoia after PTSD treatment. A recent case series with 12 patients with varying affective and psychotic disorders found that Imagery Rescripting, had a positive effect on hallucinations (37). This method was included in the TF-CBT manual used in the present study. All three patients had a subjective experience of benefitting from treatment, and the therapists also experienced that their patients profited from treatment. Overall, patients found treatment demanding but worthwhile and meaningful. Results from this study therefore contribute to the growing amount of literature that indicates that it is possible to undertake TF-CBT with patients with PTSD and co-morbid psychosis (11, 14, 15).

### 4.2 The usefulness of the TF-CBT protocol

The therapists found that it was possible to work with patients using the flexible manual and all patients seemed to profit from a combination of psychoeducation and coping strategies directed at PTSD symptoms. All methods in the protocol were used, although they were not all used with all patients. This variation reflected the different treatment needs of the participants and flexible nature of the manual. The therapy provided was considered within a CBT framework and had a trauma focus, but there was variability in the modules selected based on patient and therapist preferences. Both patients and therapists stated that the modular format was useful in adapting the treatment to the specific needs of each patient who presented with a unique constellation of challenges and symptom expressions. This modular approach may allow the tailoring of treatment to individual needs, but it requires the therapists to be competent in a range of approaches and clinical areas.

A number of these studies found that therapy, including direct processing of the trauma with Prolonged Exposure or EMDR, was superior to a waiting list (7, 20, 34). Other studies have shown that CBT without direct trauma processing was superior to a simple control treatment (16, 18, 38, 39). Some trauma-focused therapies for psychosis or serious mental illness emphasize cognitive restructuring with promising results (16, 18, 19, 40, 41) while others emphasize trauma exposure (20, 22, 34). A recent meta-analysis found that treatments including trauma exposure may be more effective for PTSD in patients with severe mental illness (24). Conversely, it has been demonstrated in several trials on Cognitive Processing Therapy for PTSD that trauma exposure may not be an essential ingredient of PTSD treatment (40, 42). The results from the current study contribute to these findings by suggesting that both direct and indirect trauma work might have beneficial effects. Direct trauma exposure was not used in the case of Heather, as the therapist and patient prioritized other modules. However, nothing in the case indicated that trauma exposure could not have been applied, and this choice might partially explain, why this patient did not improve on re-experiencing symptoms. As the case study was not designed to evaluate the effectiveness of the CBT intervention, it is not possible to make a definitive statement about the effect of treatment although it suggests that the intervention could be helpful for this group.

The decision whether to adapt the PTSD protocol for patients with psychosis as we did or to use a standard protocol is open to debate. Several studies have been published on adapted protocols including those of Rosenberg and Mueser (16, 18, 19, 39), the TRIPP protocol by Bendall (43) and a more recent study on EMDR for psychosis (44). Other studies have used a standard protocol (20, 34). We chose to adapt the TF-CBT protocol with regards to length, flexibility and focus on engagement, promoting control, relaxation techniques and case formulation in accordance with adaption made to other protocols, as there is currently no single international standard TF-CBT protocol.

It is noteworthy, that the complexity of symptoms and issues experienced by the patients in this study meant that the treatment required the utilization of more resources by the therapist compared to standard PTSD treatment with patients with no co-morbidity. These resources included the coordination with a range of inpatient and outpatient services, selection and timing of different therapeutic techniques and frequent participation in supervision. Additionally, therapists need to be highly skilled in flexible use of the manual to address patient needs.

### 4.3 Acknowledgement of methodological constraints

The case descriptions allowed detailed information about the individual issues and the course of treatment to be collected, which can directly inform clinical practice (45) Conversely, this type of design leads to a small sample size and limits generalizability of findings.

The screening and inclusion process that identified patients eligible to participate in this case-series highlighted several challenges, which could have implications for conducting larger-scale trials within this population. Participants were characterized by highly complex psychopathology, often accompanied by other co-morbid disorders including substance abuse and periods of symptom exacerbation making therapeutic treatment difficult. While there is a high prevalence of trauma and PTSD in patients with psychosis, many struggle with additional challenges in the form of cognitive difficulties or thought disorder, frequent changes in medication or regular hospitalizations, which can disrupt the continuity of therapy.

Another issue to consider when evaluating the feasibility of this intervention, is the inclusion criteria of this study, which could be considered too restrictive or stringent. It is noteworthy, that in addition to the 43 patients, who screened eligible according to inclusion criteria, a further 22 patients would have been deemed eligible, if the study had accepted clinically significant PTSD symptoms on two instead of three of the ICD-10 symptom clusters (1) re-experiencing, 2) avoidance and 3) hypervigilance and high arousal). Furthermore, when the 14 patients eligible for further assessment were evaluated with CAPS for PTSD, less than 50% of this group fulfilled the criteria for a PTSD- diagnosis, although all 14 had significant clinical symptoms. Given the relatively small scope of this study it is difficult to conclude if this pattern is representative this population in all mental health services.

The variability in patients’ backgrounds, symptoms, and external stressors complicates the interpretation of results, although most studies in the field have had broader diagnostic criteria, including patients with all types of severe mental illness (16, 20, 21, 41). It reflects a realistic picture of the target population in a community mental health context, where co-morbidity is the rule rather than the exception. However, it makes it very difficult to identify the effective treatment elements. While treatment fidelity was monitored, the flexible manual could lead to variations in application, affecting outcome consistency and reliability. The significant external stressors and life events during the treatment period make it difficult to evaluate therapy effects. However, this is most likely a challenge in most studies given that this case-series reveals that it is a common condition in community mental health patients with severe mental disorder and can be handled with randomization in a larger sample. Long-term follow-up is necessary to understand whether effects of treatment are sustained. Follow-up was attempted undertaken in this studybut due to poor attendance it was not possible to gain meaningful data. Finally, cognitive impairments are common symptoms in psychosis and chronic PTSD and can also be a side effect of psychosis treatment such as electroconvulsive therapy and antipsychotics. Cognitive impairment can affect the participants’ engagement and recall, impacting on therapy effectiveness and self-reported outcomes. This issue was likely to be the case with patient, Dorris. There are also limitations with this study as it examined only three patients seen in community mental health services, limiting the generalizability of findings.

One potential solution to ensure that people experiencing significant PTSD symptoms have access to treatment is to broaden the inclusion criteria, which a number of studies have done (7, 16, 20, 34). Although, this broader inclusion criteria could lead to a more heterogeneous sample with even more complex treatment needs. Given the resource constraints in public mental health care and the heterogeneity of the patient group without the strict inclusion criteria, a stepped care model may be relevant, where participants could firstly receive a psychoeducational intervention to promote understanding and management of symptoms (Step 1) followed by a more specialized treatment with direct trauma exposure (Step 2) for those people who did respond sufficiently after Step 1 and are deemed likely to profit from a more intensive intervention. While this approach has been tried in several clinical settings with promising findings (39, 43), there is a lack of controlled trials to demonstrate its effectiveness. A stepped care approach would make the treatment accessible to a broader and less diagnosis specific group of patients with SMI and PTSD, thus increasing the target population and making it possible for less specialized staff to undertake the intervention.

## 5 Conclusion

The aim of the current study was to evaluate the feasibility of TF-CBT treatment of patients with comorbid PTSD and psychosis. Acknowledging the limits of our study design, results from the case series suggested that individual TF-CBT could be meaningfully implemented and lead to a decrease in PTSD symptomology. TF-CBT was also deemed acceptable with relatively few negative effects. The complexity and variation in psychopathology within these cases was reflected in the utility of a flexible modular manual where a range of techniques were used to reflect the different treatment needs of patients. Further research is required to determine the efficacy of this intervention and examine issues concerning the identification and recruitment of suitable patients in relation to treatment needs and treatment intensity.

## Data Availability

The datasets for this article are not publicly available due to concerns regarding participant/patient anonymity. Requests to access the datasets should be directed to the corresponding author.
